# Microcrystalline tyrosine as a novel depot-forming agent in venom immunotherapy: a pre-clinical evaluation in bee-venom allergic mice

**DOI:** 10.3389/falgy.2026.1840453

**Published:** 2026-05-18

**Authors:** Marta Paolucci, Agathe Duda, Lara Šošić, Louise Wallace, Simon J. Hewings, Murray A. Skinner, Thomas M. Kündig, Matthias F. Kramer, Pål Johansen

**Affiliations:** 1 Dept. Dermatology, University of Zurich, Zurich, Switzerland; 2 Dept. Dermatology, University Hospital Zurich, Switzerland; 3 Allergy Therapeutics plc, Worthing, United Kingdom

**Keywords:** adjuvant, allergen immunotherapy, aluminium, anaphylaxis, hymenoptera, microcrystalline tyrosine, mouse model, venom

## Abstract

**Background:**

Venom immunotherapy (VIT) with aqueous venom extracts is a standard treatment for severe insect venom allergies. In other fields of allergen immunotherapy (AIT), depot adjuvants have been used for decades, with benefits for both safety and efficacy. Biodegradable microcrystalline tyrosine (MCT), is well-established in AIT, and has proven both safe and effective through sustained release and adjuvancy. The objective of the current study was to evaluate MCT as a depot-forming agent in VIT using a murine model of bee venom allergy.

**Materials and methods:**

Mice were sensitised with bee venom extract and then received subcutaneous immunotherapy (SCIT) with 10, 50, or 100 µg aqueous venom extracts or venom formulated with MCT or aluminium hydroxide (alum) as depot-forming agents. Systemic reactions upon VIT and challenge were assessed by measuring body temperature, while antigen-specific IgE and IgG responses were analysed by ELISA. Mast cell degranulation was evaluated by serum MCPT-1 levels.

**Results:**

VIT with MCT significantly improved survival, reduced body temperature changes, and promoted robust IgG1 and IgG2b responses. While antibody responses were comparable between MCT and alum at higher VIT doses (50 and 100 µg), MCT also induced significant IgG responses at lower doses (10 µg), indicating enhanced sensitivity of the immune response to MCT. MCT-treated mice further exhibited reduced mast-cell degranulation compared to untreated controls, consistent with a reduced risk of anaphylaxis.

**Conclusion:**

VIT with bee venom extract and MCT enabled safe and effective VIT by promoting protective IgG responses and reducing systemic reactions. These findings further support the evaluation of MCT as a complement to aqueous allergen extracts in VIT for human use.

## Introduction

Venom immunotherapy (VIT) is an established and highly effective treatment for patients with severe allergies to insect venom, including those from *Vespula* wasps and *Apis mellifera* honeybees. By gradually increasing exposure to venom allergens, VIT induces immune tolerance and substantially reduces the risk of severe allergic reactions, including anaphylaxis, upon future stings. While VIT provides robust and long-term protection (1, 2), further optimisation of allergen delivery remains desirable and may improve tolerability and patient compliance (3–5).

One strategy to improve allergen delivery is the use of depot-forming agents, long practiced in subcutaneous immunotherapy (SCIT), most commonly aluminium hydroxide (alum). Comparisons between alum-adsorbed and aqueous VIT preparations point towards potential safety benefits for depot products, although these studies are limited by confounding factors, e.g., highly vs. less purified, and in-patient rush vs. out-patient conventional posology (6, 7). A recent study examining “purified”, “highly purified”, and “non-purified” *Hymenoptera* venom extract demonstrated that such classifications do not necessarily translate into clinically meaningful differences in treatment effectiveness or systemic adverse events (AE) (8). As all extracts are manufactured according to European *Pharmacopoeia* standards, therapeutic performance appears largely comparable across formulations. Aqueous naturally composed VIT has been shown to provide near-complete protection in both wasp and bee venom allergic patients, a level of efficacy that is difficult to improve upon (9). At the same time, long-term administration of alum-adsorbed allergen extracts has raised concerns regarding cumulative aluminium exposure, increasing the patient's interest in alternative adjuvant systems that reduce aluminium burden without compromising therapeutic outcomes. In line with this, current guidelines advise against alum-adjuvanted extracts for maintenance doses exceeding 100 µg, which may be required in venom allergic patients with certain risk factors (1).

The traditional aqueous venom extracts are effective in initiating immune responses but lack controlled allergen release, which may contribute to systemic reactions. Depot-forming agents can create sustained allergen release, thereby limiting bolus exposure, reducing dose-dependent AEs, and potentially decreasing injection frequency (7, 10), all while maintaining the immunogenicity of the allergen. However, alum-based depot formulations may preferentially promote T-helper type 2 (Th2)-biased immune activation (11), similar to low-dose and non-adjuvanted allergens, which may limit the induction of long-term tolerance that is rather Th1 associated (12). Alum has also been associated with persistent local reactions, and concerns regarding aluminium accumulation remain particularly relevant in the context of life-long VIT (13–16). Beyond immunological considerations, recent research has highlighted that the presence of alum influences patient preferences. Patient-reported preferences have recently shown to prioritise long-term safety attributes, especially avoidance of foreign material accumulation, such as alum, emphasising the need to consider such factors when developing and selecting VIT formulations that better align with patient expectations and safety concerns (3).

These limitations have stimulated interest in alternative depot adjuvants for VIT and for allergen immunotherapy (AIT) in general (12, 17, 18). Certain adjuvants can improve allergen recognition and uptake by antigen presenting cells, such as dendritic cells and macrophages, by presenting allergens in particulate form. This facilitates more efficient antigen processing and modulates the immune response quality, which may promote a more balanced Th1/Th2 immune profile, thereby facilitating long-term tolerance.

Microcrystalline tyrosine (MCT) has emerged as a promising alternative excipient in this context (19). MCT is biodegradable and biocompatible, and its use in drug delivery systems is well established due to its ability to form stable complexes with proteins. When used in AIT and vaccination, MCT has been shown to facilitate balanced immune responses with a shift toward Th1 immunity (11, 20), which is associated with the induction of IgG antibodies and suppression of IgE-mediated allergic reactions including anaphylaxis. Moreover, MCT provides a depot effect that supports allergen persistence at the injection site, potentially enhancing immunogenicity while allowing for fewer injections and enhanced patient compliance.

Despite these advantages, the introduction of novel adjuvants or depot-forming agents into VIT requires careful evaluation. Given the inherent risk of systemic reactions during VIT, a possible change in excipients must not introduce new safety risks. In this study, we therefore employed a murine model of venom-induced anaphylaxis to assess the immunogenicity and safety of MCT-adjuvanted bee venom allergen extracts. We compared these extracts with aqueous formulations and alum-adjuvanted preparations commonly used in clinical practice.

## Materials and methods

### Materials

Bee venom extract (Venomil, 550 µg) and MCT (40 mg/ml) were provided by Allergy Therapeutics (Worthing, United Kingdom). The venom extract was freshly prepared by dissolving it in physiological phosphate-buffered saline (PBS) to a final concentration of 1 mg/ml. Aluminium hydroxide (Alhydrogel 2%) was purchased from InvivoGen through Thermo Fisher Scientific (Basel, Switzerland). The major bee venom allergen phospholipase A2 (PLA2; Api m 1) was purchased from Sigma-Aldrich (#P9279; Buchs, Switzerland).

### Animals

Female BALB/cOlaHsd (H-2^d^) and C3H/HeNHsd (H-2^k^) mice were purchased from Envigo (Horst, The Netherlands). Animals were housed and all experiments performed under specific pathogen free conditions at the Laboratory Animal Services Center (LASC), University of Zurich. Mice were kept in individually ventilated cages at 21°C with a 12–12 h light-dark cycle and *ad libitum* access to water and chow. All experimental procedures were approved by the Cantonal Veterinary Office Zurich and performed in accordance with Swiss animal legislations (authorization ZH 095/2022) and the ARRIVE 2.0 guidelines (21).

### Sensitisation

Mice were sensitised with alum-based formulations of bee venom extract. Sensitisation solutions were prepared fresh on the day of use and contained 10 µg/mL extract in PBS. The final formulation contained 33% Alhydrogel corresponding to 0.66 mg alum per dose. Mice were sensitised once weekly for six weeks with 1 µg venom extract per injection by administering 100 µL of the preparation subcutaneously into scruff of the neck. Tail vein blood was collected before and after the sensitisation phase, and sera were analysed for allergen-specific antibodies. Sensitisation was confirmed by PLA2-specific IgE ELISA (see below).

### Venom immunotherapy (VIT)

Three weeks after the final sensitisation, mice received VIT with 10–100 µg venom extract formulated in PBS alone, PBS with alum (0.66 mg), or PBS with MCT (2 mg). After rigorous vortexing (>1 min), preparations were left at room temperature for 1 h to allow complete allergen adsorption. VIT was administered subcutaneously into the neck scruff (100 µL). Treatments were given three times at one-week intervals. After the third VIT, core body temperature was measured rectally 30 min post-injection using a digital Thermalert TH-5 thermometer with a RET-3 probe (Physitemp Inc., Huron, NJ) to detect potential systemic allergic reactions as described (22, 23). A sensitised-only group served as control. Tail vein blood was collected before and after VIT and sera analysed for PLA2- and bee venom extract-specific antibodies.

### Allergen challenge test for analysis of anaphylaxis

Three weeks after last of three VIT sessions, the mice were challenged by an intraperitoneal injection of 100 µL of 30 µg bee venom extract in PBS. Core body temperature was measured rectally with the digital thermometer before the challenge and then measured at regular intervals for 2–3 h thereafter. A time-temperature curve was plotted and the area under curve (AUC) was calculated by integrating the difference between baseline temperature and the measured body temperature over 0–120 or 0–150 min:

$$\mathrm{AUC}=\overset{t_{n}}{\underset{t_{0}}{\int }}\;[T(t)+T_{\mathrm{baseline}}]\mathrm{dt}$$

Because temperature decreases during anaphylaxis, this produces exclusively positive AUC values; larger AUC indicates a stronger anaphylactic response. All calculations were done in GraphPad Prism v8.0.0 from GraphPad Software (La Jolla, US-CA).

The primary endpoint for the effect of VIT was the reduced AUC after VIT as compared to sensitised but untreated mice. The effect of VIT was also monitored by scoring clinical symptoms of allergy, e.g., self-grooming, piloerection, hunched posture, reduced mobility, and reaction to handling and sounds, as well as the time to reach a body temperature of 34°C plotted in a Kaplan–Meier curve. Body temperature lower than 30°C was considered as the endpoint of the experiment and criteria of termination. One week after the challenge with bee venom extract, mice were rechallenged with 30 µg PLA2 in PBS and the body temperature and anaphylaxis were monitored as described above.

### Analysis of serum antibodies by ELISA

Sera were analysed for antibodies specific for the major bee venom allergen PLA2 using an established ELISA method. For IgG measurements, Maxisorb 96-well plates were coated with PLA2 (5 µg/ml) in a carbonate buffer at pH 9.4. For IgE, the plates were coated with 3 g/ml of rat anti-mouse IgE heavy chain antibody (BioRad, #MCA419; Hercules, US-CA). After overnight incubation at 4°C and blocking with 2.5% non-fat-dried skimmed cow milk in PBS-Tween20 (PBSTM), serial dilutions of mouse serum in PBSTM were added and plates incubated for 2 h. Biotinylated anti-murine antibodies (BD Pharmingen, Basel, Switzerland) were then added for IgG analysis and biotinylated PLA2 was added for IgE analysis. The plates were then incubated with Streptavidin conjugated HRP and developed with TMB substrate from BioLegend (Koblenz, Germany). Each incubation step was followed by washing the plates with PBST. The colorimetric enzyme reaction was stopped with 2N sulfuric acid and the absorbance read at 450 nm using a BioTek ELx808 microplate reader (Luzern, CH). The antibody titres were defined as the last serum dilution giving an ELISA OD higher than the mean of naïve sera plus thrice the standard deviation of the mean.

Alternatively, sera were analysed for IgG1 and IgG2b antibodies specific to bee venom extract using a modified ELISA protocol as described above. MaxiSorp 96-well plates were coated with bee venom extract (25 µg/mL) in carbonate buffer (pH 9.4). Following blocking, serial dilutions of mouse sera were applied and antibody binding was detected as described for PLA2-specific ELISA. Results are presented as titration curves, plotting ELISA optical density (OD) against the reciprocal serum dilution.*in vitro* assessment of mast cell protease 1 (MCPT-1) and cytokines.

To assess if the allergen challenge was also associated with systemic allergic reactions as measured by release of mast cell protease 1 (MCPT-1), sera were collected 90 min after the challenge and measured using a commercial MCPT1 kit from Thermo Fisher Scientific according to the instructions provided by the supplier. Alternatively, the post-challenge sera were analysed for the content of TNF-α and IL-6 cytokines using coating antibodies from BioLegend (# 510801) and Invitrogen (# 14-7061-81), respectively, and as described by the provider.

### Endpoints

The primary study endpoint was the severity of anaphylaxis following allergen challenge, quantified by the area under the time-temperature curve (AUC). Secondary endpoints included (i) clinical anaphylaxis scores, (ii) time to reach a core temperature of 34°C, (iii) serum MCPT-1 levels following challenge, and (iv) allergen-specific IgE and IgG antibody titres before and after VIT. Safety-related endpoints included monitoring for VIT-induced systemic reactions and human endpoint temperature (<30°C).

### Statistics

Statistical analysis was done with GraphPad Prism v7.02. Two treatment groups were compared using non-parametric, 2-tailed Mann–Whitney *U*-tests. More groups were compared using Kruskal–Wallis tests with Dunn's *post hoc* test for multiple comparisons. Time-temperature curves were integrated using the baseline temperature for the calculation of the area under curve (AUC). Two-way parametric ANOVA was applied to test the statistical differences between the different treatment groups. Prior to this, normally distributed data was confirmed using the D'Agostino and Pearson normality test. The probability of survival was defined as the time to reach a core temperature of 34°C within the temperature-monitoring period and was measured using a Mantel-Cox logrank test with Holm-Sidak's multi-comparison test. IgE, IgG1, and IgG2 antibodies are presented as titres and as geometric means with geometric standard deviations for individual mice. Significant differences were analysed by one-way ANOVA assuming lognormal distributions, with Tukey corrections for multiple comparisons, and were annotated with asterisks: *p* < 0.05: *; *p* < 0.01: **; *p* < 0.001: ***; *p* < 0.0001: ****.

## Results

### Establishment of a murine model of bee venom-induced anaphylaxis

To establish a robust model of allergic anaphylaxis, we systematically tested different mouse strains, sensitisation regimens, and challenge doses (data not shown). Both H-2^k^ C3H/HeNHsd and H-2^d^ BALB/cOlaHsd mice were evaluated, with sensitisation using bee venom allergen extract (0.1–10 µg) administered 2–6 times, with or without alum. While intraperitoneal sensitisation was ineffective in inducing anaphylaxis upon subsequent challenge, subcutaneous administration produced reproducible systemic allergic reactions. High sensitisation doses (5–10 µg) were protective rather than anaphylactic, and fewer injections (≤4) failed to generate robust responses. Challenge doses of 5–10 µg were insufficient to provoke strong reactions. Overall, C3H/HeNHsd mice sensitised subcutaneously with 1 µg allergen over six weekly injections and challenged with 30 µg bee venom extract exhibited the most consistent and robust anaphylactic responses, assessed by body temperature drop and clinical signs. In experiments performed in the method development, 20 out of 23 mice (87%) sensitised with six weekly subcutaneous injections of bee venom extract reacted to a challenge with hypothermia and clinical signs of anaphylaxis (data not shown). This optimised protocol was then used to evaluate the safety and efficacy of VIT.

### Enhanced protection from anaphylaxis at higher bee venom extract doses

**Figure 1:**
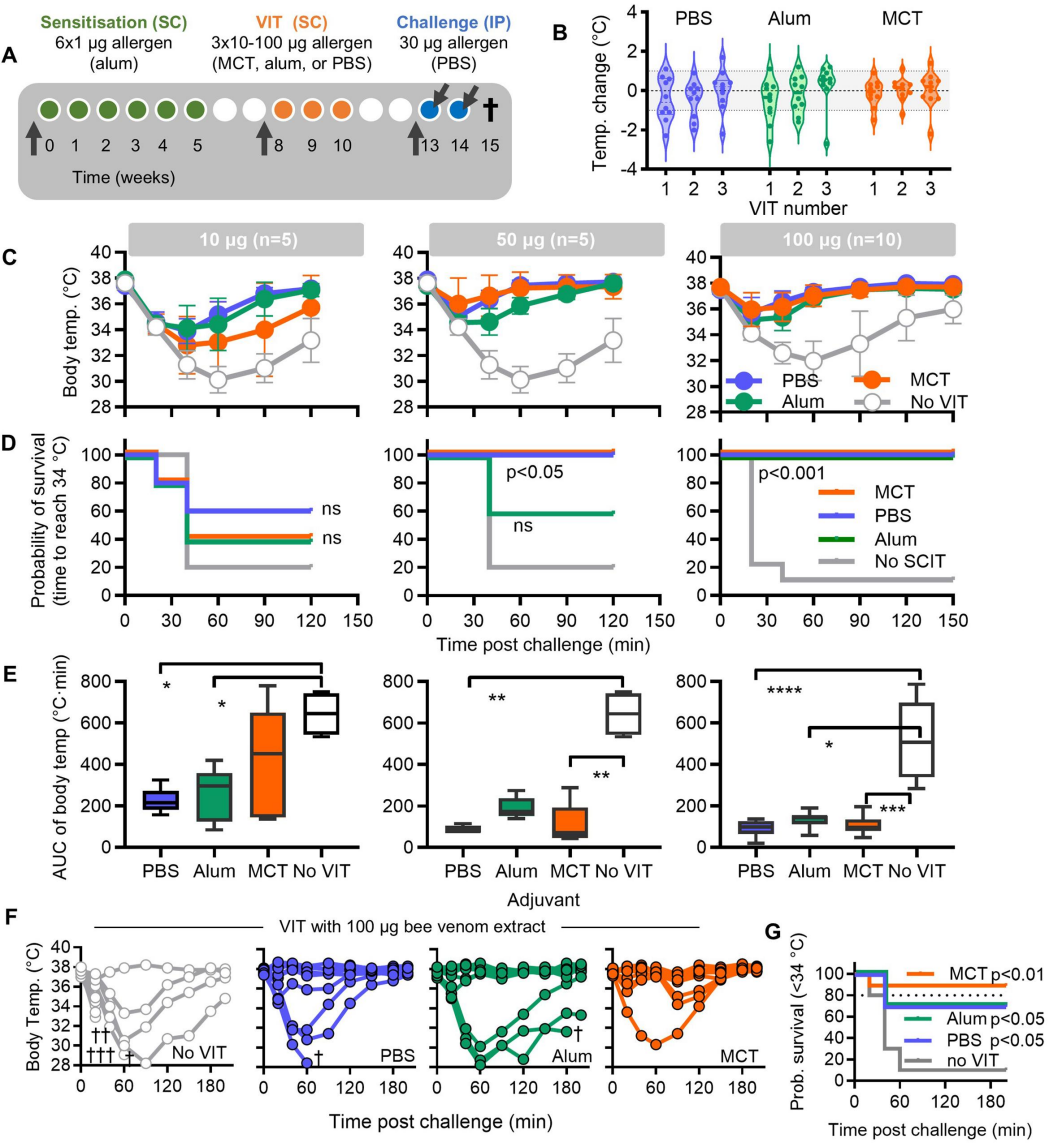
Venom immunotherapy with bee venom extract in C3H mice. **(A)** Scheme showing the subcutaneous (SC) sensitisation with bee venom extract with alum (green; weeks 0-5), VIT with 10, 50 or 100 µg bee venom extract formulated with MCT, alum, or PBS buffer (orange; weeks 8-10), and allergen challenge tests (blue) with bee venom extract (week 13) and major bee venom allergen PLA2 (week 14). Arrows indicate time point for blood collection. **(B)** Change in body temperature measured 30 min after the 1st, 2nd, and 3rd SCIT sessions in mice receiving 100 µg bee venom extract formulated with PBS, alum, or MCT. Each dot represents an individual mouse; violin plots depict distribution and median values. Hypothermia was defined as a ≥ 1°C decrease from baseline (dotted lines). **(C)** Core body temperature (BT) measured 120-150 min after challenge with allergen extract (week 13) and as a function of VIT adjuvant. Control mice (grey line, white symbols; No VIT) were sensitised but not treated. VIT with PBS, alum, or MCT are indicated with blue, green, or orange symbols and lines. **(D)** The efficacy of VIT as measured by Kaplan–Meier curves in which incidents are defined as time to reach a BT of 34°C after challenge with allergen extract. **(E)** The efficacy of VIT as measured by Area Under Curve (AUC) of the time-temperature integral. **(F)** Core BT measured 210 min after challenge with PLA2 (week 14) and as a function of VIT adjuvant. Each temperature line represents one individual mouse. Mice that met a criterium for termination are indicated with a cross. **(G)** The efficacy of VIT as measured by Kaplan–Meier curves in which incidents are defined as time to reach a BT of 34°C after challenge with PLA2. *P* values indicate statistical difference with untreated mice.

In the established mouse model of insect venom allergy (Figure 1A), venom-sensitised mice received VIT with 10 µg (*n* = 5), 50 µg (*n* = 5), or 100 µg (*n* = 10) bee venom extract, administered as an aqueous solution (PBS) or formulated with alum or MCT as depot. The experiments were performed in two parts: one including the 10 µg and 50 µg doses, and a second including the 100 µg dose.

Given the potential for systemic allergic reactions following VIT, we monitored both body temperature changes and clinical signs of allergy after VIT with 100 µg of bee venom extract, either in aqueous or depot formulations. Body temperature was measured 30 min post-injection, and clinical symptoms, including hunched posture, piloerection, nasal discharge, orbital tightening, and general activity, were recorded. After the first VIT session, piloerection was observed in 7 out of 30 mice (23%), with 5 mice (50%) in the aqueous VIT group (results not shown). In contrast, only 1 mouse (10%) in the alum group and 1 mouse (10%) in the MCT group exhibited piloerection. No clinical reactions were noted after the second and third VIT sessions. In terms of body temperature, hypothermic reactions (defined as a ≥ 1°C drop compared to baseline) were observed in 4 mice (40%) in the PBS group, 3 mice (30%) in the alum group, and 1 mouse (10%) in the MCT group following the first VIT (Figure 1B). After the second VIT session, hypothermia was seen in 3 mice (30%) from the PBS group, 2 mice (20%) from the alum group, and 1 mouse (10%) from the MCT group. Only one mouse per group showed hypothermic reactions after the third VIT.

Body temperature was monitored following bee venom extract challenge to assess systemic reactions across treatment groups compared with sensitised but untreated mice (Figure 1C, Table 1). Baseline body temperatures were comparable across all groups, with a mean of 37.6°C (range: 36.8–38.3°C). Following challenge, body temperature decreased rapidly, with the lowest temperatures reached between 20 and 60 min depending on VIT dose and adjuvant. In the 10 µg VIT group, mean minimal temperatures were 33.9°C (32.4–34.8°C) for PBS, 34.1°C (32.7–36.1°C) for alum, and 32.8°C (30.0–35.3°C) for MCT. Animals receiving 50 µg or 100 µg VIT exhibited a less pronounced drop in temperature and faster recovery, with minimal temperatures observed around 20 min post-challenge. For the 50 µg VIT group, mean minimal temperatures were 35.0°C (34.3–35.5°C) for PBS, 34.6°C (34.1–35.4°C) for alum, and 36.0°C (34.2–38.3°C) for MCT. Corresponding values for the 100 µg VIT group were 35.7°C (34.5–38.0°C) for PBS, 34.4°C (34.5–38.3°C) for alum, and 36.5°C (34.6–38.4°C) for MCT. In contrast, sensitised but untreated controls exhibited more pronounced hypothermia, with mean minimum temperatures of 30.1°C (29.1–31.1°C) for the 10–50 µg control experiment and 32.0°C (30.2–34.5°C) for the 100 µg control experiment. All animals recovered within 2–3 h post-challenge.

**Table 1:** Key figures from the challenge assay.

VIT formulation	Min T (°C; range)^c^	Temp AUC (°C h)		Survival	
		Mean (range)	*P* value^d^	%	*P* value^e^
10 µg, PBS	33.9 (32.4–34.8)	224 (156–324)	<0.05	40	ns
10 µg, Alum	34.1 (32.7–36.1)	253 (85–420)	<0.05	60	ns
10 µg, MCT	32.8 (30.0–35.3)	408 (137–778)	ns	40	ns
50 µg, PBS	35.0 (34.3–35.5)	87 (72–114)	<0.01	100	<0.001
50 µg, Alum	34.6 (34.1–35.4)	190 (139–274)	ns	60	ns
50 µg, MCT	36.0 (34.2–38.3)	110 (43–288)	<0.01	100	<0.001
100 µg, PBS	35.7 (34.5–38.0)	92 (19–137)	<0.0001	100	<0.001
100 µg, Alum	34.4 (34.5–38.3)	135 (58–190)	< 0.05	100	<0.001
100 µg, MCT	36.5 (34.6–38.4)	108 (47–195)	<0.001	100	<0.001
Sensitised only A^a^	30.1 (29.1–31.1)	643 (534–749)	–	20	–
Sensitised only B^b^	32.0 (30.2–34.5)	531 (283–787)	–	20	–

Minimal body temperature (°C) recorded after the challenge is reported along with the observed range. The probability of “survival” (%) reflects the likelihood of maintaining a body temperature above 34°C over the measured time period, based on time to reach this threshold after the challenge. Baseline body temperatures were comparable across all groups, with a mean of 37.6°C (range: 36.8–38.3°C).

a Sensitisation control (no VIT) for experiments with 10 µg and 50 µg challenge.

b Sensitisation control (no VIT) for experiments with a 100 µg challenge.

c The range indicates the individual mice with the lowest and the highest minimum body temperature post-challenge.

d Kruskal–Wallis analysis with Dunn's multiple comparisons test, with each treatment compared with either of the two sensitised controls (A or B).

e Kaplan–Meier analysis with Holm-Šídák's multiple comparisons test, with each treatment compared with either of the two sensitised controls (A or B).

The probability of survival, defined as the time to reach a core temperature of 34°C, increased in VIT-treated mice in a dose-dependent manner (Figure 1D, Table 1). In the 10 µg VIT group, survival was 40% for PBS and MCT and 60% for alum, compared with 20% in sensitised, untreated controls (non-significant). At the 50 µg VIT dose, survival improved significantly for PBS and MCT (100%) but not for alum (60%) relative to untreated controls (20%; *p* < 0.05). VIT with 100 µg extract resulted in 100% survival across all formulations, compared with <20% in untreated controls (*p* < 0.001).

The benefit of VIT was further confirmed by analysis of the time–temperature integral (area under the curve, AUC; Figure 1E). Across all doses and formulations, VIT reduced AUC values relative to untreated controls. This reduction reached statistical significance for all 100 µg VIT groups (PBS: *p* < 0.0001; alum: *p* < 0.05; MCT: *p* < 0.001), for PBS and MCT at 50 µg (*p* < 0.01), and for PBS and alum at 10 µg (*p* < 0.05).

One week after the initial challenge with whole venom extract, mice were re-challenged with 30 µg of the major allergen PLA2, and body temperature was monitored as described. Hypothermic responses were generally stronger than those observed with whole venom extract. For mice receiving 100 µg bee venom VIT, 8 out of 40 mice (20%) had to be terminated early due to severe hypothermia (Figure 1F). Termination criteria were met in 6 of 10 untreated mice (60%), compared with 1 of 10 mice in the PBS and alum VIT groups (10% each). Kaplan–Meier analysis (Figure 1G) of the time to reach 34°C demonstrated a significant survival benefit for VIT-treated mice: 70% survival for PBS and alum (*p* < 0.05) and 90% survival for MCT (*p* < 0.01). Due to early terminations in some animals, an AUC analysis for this PLA2 challenge could not be performed. Similar results were obtained for mice receiving 10 and 50 µg bee venom VIT (data not shown).

### VIT effectively elicits allergen-specific IgG antibodies

**Figure 2:**
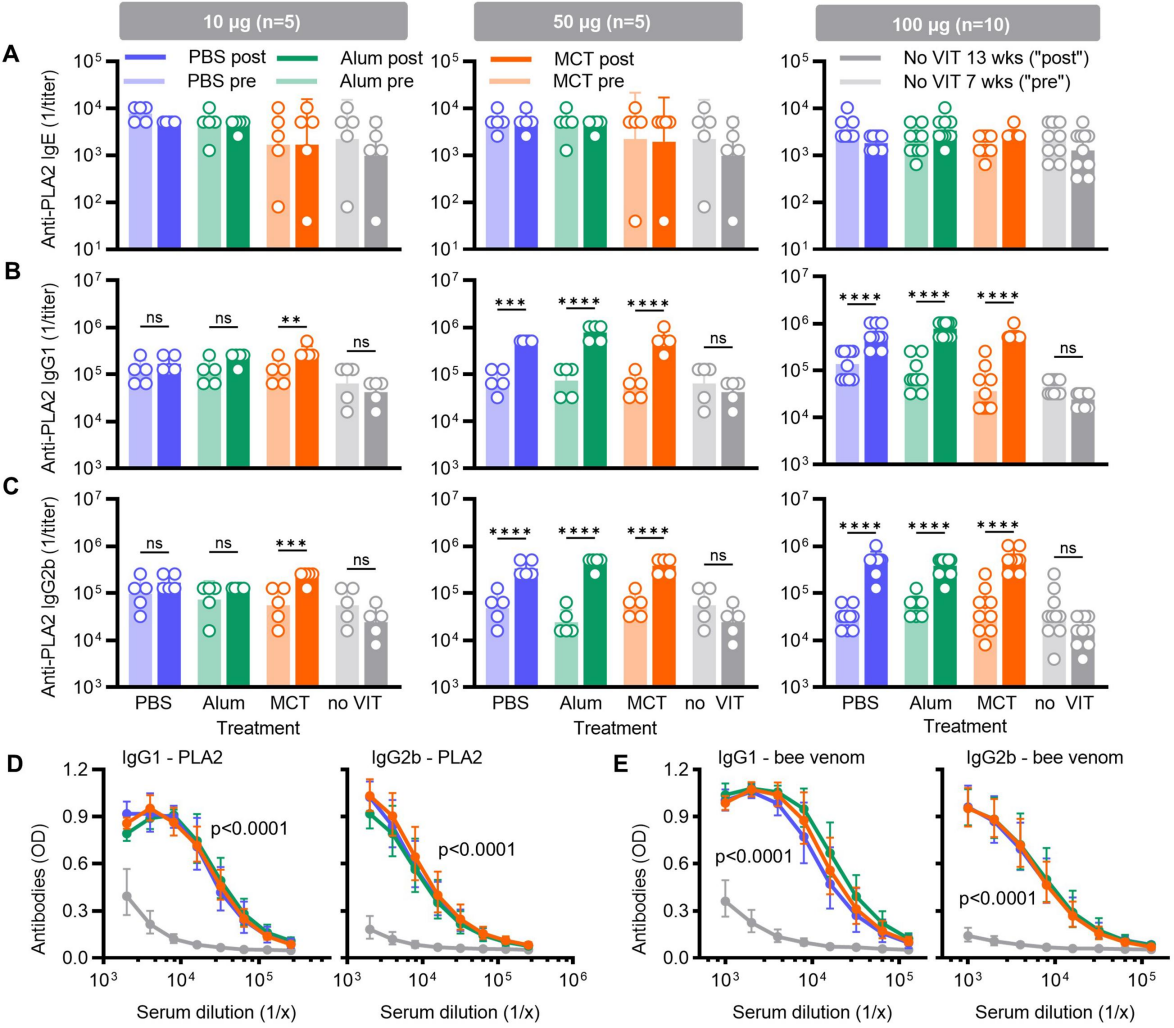
Antibody responses to VIT with bee venom extracts in mice. Mice were sensitised and VIT treated with bee venom extract as described in Figure 1A. Before (7 weeks, light-colour bars, “pre” VIT) and after (13 weeks; dark-colour bars, “post” VIT) immunotherapy with 10, 50, or 100 µg bee venom extract formulated with PBS (blue), alum (green), or MCT (orange), blood was collected and serum analysed for allergen-specific IgE **(A)**, IgG1 **(B)**, and IgG2b **(C)** reciprocal titres. Sensitised but otherwise untreated mice (grey) were analysed at the same timepoints. The bars illustrate geometric means of reciprocal titres, error bars show geometric standard deviations, and open circles show results from individual mice. The statistical analysis indicates if determined *p* values are >0.05 (ns), <0.05 (*), <0.01 (**), <0.001 (***), and <0.0001 (****). **(D)** Serum titration curves of PLA2-specific IgG1 and IgG2b antibodies in sensitised mice or in mice treated with 100 µg bee venom extract formulated in PBS, alum, or MCT at 13 weeks. **(E)** Serum titration curves of bee venom extract-specific IgG1 and IgG2b antibodies in the same treatment groups. Titration curves are presented as ELISA OD plotted against the reciprocal serum dilution and *p* value indicate difference between any treated groups and untreated mice.

Antigen-specific IgE, IgG1, and IgG2b responses were measured before and after VIT across all three dose groups (10 µg, 50 µg, and 100 µg). Regardless of VIT dose or formulation, PLA2-specific IgE responses remained largely unchanged pre- and post-VIT (Figure 2A).

At the 10 µg VIT dose, IgG1 and IgG2b levels were not substantially altered in the PBS- or alum-treated groups. In contrast, MCT-depot VIT induced a nearly three-fold increase in PLA2-specific IgG1, with the geometric mean rising from 128,000 to 307,200 (*p* < 0.01), and a four-fold increase in PLA2-specific IgG2b, from 73,600 to 230,400 (*p* < 0.001; Figures 2B,C).

At the 50 µg VIT dose, all formulations significantly increased PLA2-specific IgG1 and IgG2b titres. The most pronounced effect on IgG1 was observed with alum (9-fold increase, from 89,600 to 819,200; *p* < 0.0001) and MCT (9-fold, from 64,000 to 563,200; *p* < 0.0001). For IgG2b, all formulations significantly boosted antibody responses by 6- to 16-fold (*p* < 0.0001).

Finally, VIT with 100 µg bee venom extract induced robust increases in both IgG subclasses. IgG1 levels rose 4- to 9-fold, and IgG2b levels increased 7- to 15-fold across the three formulations (*p* < 0.0001; Figures 2B,C). The corresponding serum titration curves were comparable between all three VIT formulations (Figure 2D).

To further assess whether different treatment modalities affected antibody responses beyond the major allergen PLA2, PLA2-specific findings were complemented by measurements of IgG1 and IgG2b antibodies against whole bee venom extract in mice treated with 100 µg VIT. As shown in Figure 2E, sera from VIT-treated mice exhibited markedly higher titration curves compared with sensitised but untreated controls, whereas only minor differences were observed between the three VIT formulations. Bee venom extract-specific IgG1 titres reached 256,000 in the PBS group, 446,000 in the alum group, and 362,000 in the MCT group, compared with 15,000 in untreated controls. While differences between VIT groups were not statistically significant (*p* > 0.05), all treated groups differed significantly from untreated controls (*p* < 0.0001). Comparable results were obtained for IgG2b, with nearly identical titration curves and antibody titres across all VIT-treated groups, again clearly separated from untreated controls (Figure 2E).

### VIT reduces mast cell degranulation

Mast cell activation and degranulation following allergen challenge were assessed by measuring serum levels of MCPT-1 in mice that had received VIT with 100 µg bee venom extract formulated in PBS, alum, or MCT. Mice were challenged either with whole bee venom extract or with the major allergen PLA2.

**Figure 3:**
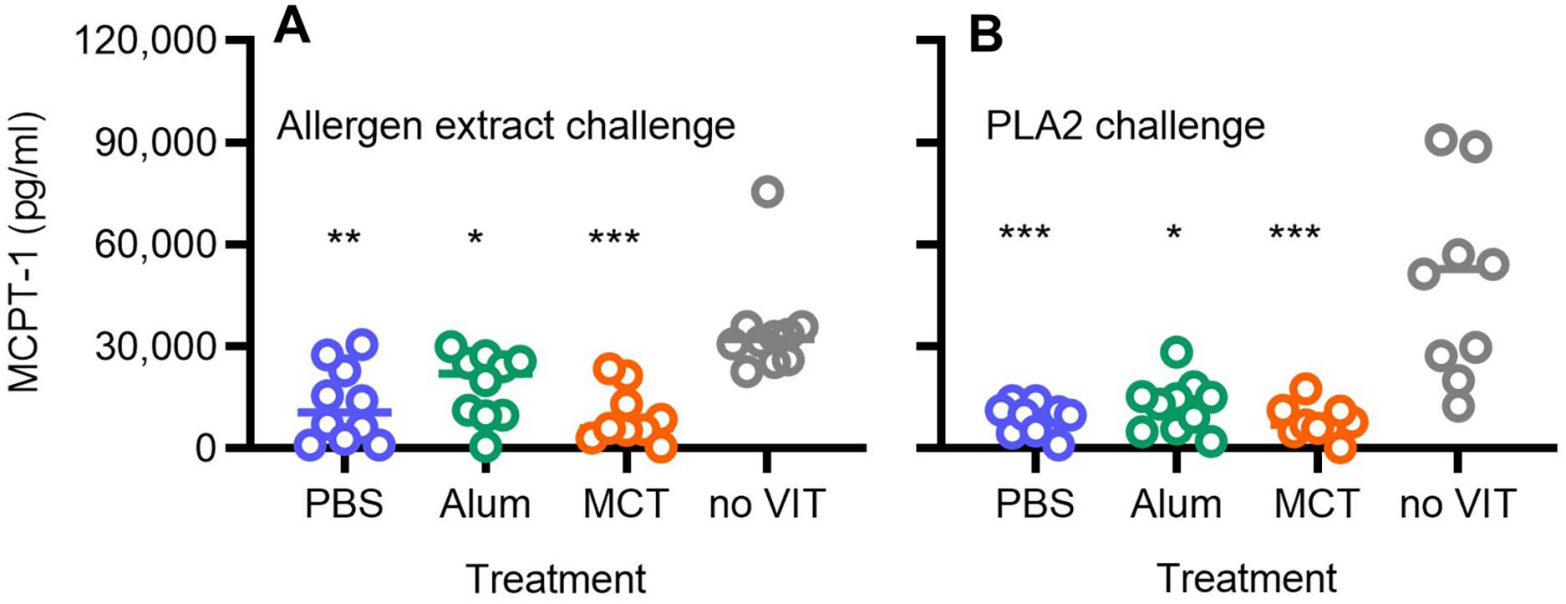
Release of MCPT-1 after allergen challenge of allergic mice. Mice were sensitised and VIT treated with bee venom extract as described in Figure 1A. Ninety minutes after the challenge with bee venom extract **(A)** and PLA2 **(B)**, venous blood was drawn and the sera analysed for the content of MCPT-1 by ELISA and as a function of treatment. The open circles show results from individual mice. The statistical analysis indicates if determined *p* values are <0.05 (*), <0.01 (**), or <0.001 (***) as compared to sensitised but not treated mice (no VIT, grey colour).

Following challenge with bee venom extract, VIT-treated mice exhibited a marked reduction in MCPT-1 release compared with sensitised, untreated controls (Figure 3A). Sensitised control mice showed high MCPT-1 levels, with a mean serum concentration of 35,041 pg/mL (range: 22,734–76,651 pg/mL). In contrast, VIT significantly reduced MCPT-1 levels across all formulations: 12,944 pg/mL (1,112–30,641 pg/mL) in PBS-treated mice (*p* < 0.01), 18,360 pg/mL (776–29,914 pg/mL) in alum-treated mice (*p* < 0.05), and 9,686 pg/mL (520–23,551 pg/mL) in MCT-treated mice (*p* < 0.001).

Comparable results were observed following PLA2 challenge (Figure 3B). Sensitised, untreated mice exhibited higher MCPT-1 levels, with a mean serum concentration of 56,872 pg/mL (12,553–136,991 pg/mL). VIT again significantly reduced MCPT-1 concentrations: 8,896 pg/mL (1,070–14,161 pg/mL) in PBS-treated mice, 12,727 pg/mL (2,139–26,183 pg/mL) in alum-treated mice, and 8,031 pg/mL (340–17,524 pg/mL) in MCT-treated mice.

To assess whether allergen challenge induced systemic cytokine release, serum levels of TNF-α and interleukin-6 were measured in mice treated with 100 µg bee venom extract. Both cytokines were below the detection limit in sera collected 90 min post-challenge (data not shown), which is consistent with the early time point of sampling and the known delayed kinetics of cytokine production.

## Discussion

Venom immunotherapy (VIT) is a highly effective disease-modifying treatment for Hymenoptera venom allergy (1, 2). However, systemic adverse reactions during treatment and the need for prolonged dosing regimens remain important clinical challenges (6, 7, 10). In this study, we used a murine model of bee venom–induced anaphylaxis to evaluate whether MCT can be used as a depot-forming agent for venom extracts without compromising efficacy or safety. The results demonstrate that MCT-based VIT performed comparably to both aqueous and alum-based formulations, providing protection against systemic anaphylaxis to a similar extent, while maintaining a favourable safety profile. These findings provide important insights for the potential use of MCT in VIT formulations, offering an adjuvating option that matches the established performance of alum, and supporting its potential use in improving the safety of VIT for human patients with insect venom allergies.

Efficacy was first evident at the clinical level, where VIT reduced hypothermic responses, improved survival, and decreased the overall severity of anaphylaxis following allergen challenge in a dose-dependent manner. At higher VIT doses (50 and 100 µg), all formulations provided substantial protection compared with sensitised but untreated controls. Importantly, MCT-based VIT consistently performed at least as well as aqueous or alum-based formulations, including during rechallenge with the major venom allergen PLA2, which elicited particularly strong anaphylactic responses. These findings confirm that using VIT with MCT does not impair the protective efficacy in this pre-clinical setting.

Beyond clinical readouts, we assessed mast cell activation as a functional effector endpoint by measuring serum levels of MCPT-1 after allergen challenge. MCPT-1 is a well-established biomarker of systemic mast cell degranulation and is more stable than histamine which is released in parallel to MCPT-1. MCPT-1 also correlates closely with the severity of IgE-mediated anaphylaxis in murine models (24). Sensitised, untreated mice exhibited high MCPT-1 levels following challenge with whole venom extract or PLA2, whereas VIT markedly reduced MCPT-1 release across all formulations. Notably, the reduction in mast cell effector activation observed in mice receiving MCT-based VIT was comparable to that achieved with alum-based VIT. These data indicate that MCT-formulated VIT effectively limits mast cell effector activation upon allergen re-exposure, providing mechanistic evidence of efficacy that complements the observed clinical protection. Reduced mast cell degranulation is consistent with the induction of protective immune mechanisms, such as allergen-specific IgG antibodies capable of blocking IgE-mediated activation of Fc*ε*RI-expressing cells (25). No systemic cytokine release was detected 90 min after allergen challenge. This is consistent with the kinetics of mast cell activation, as most cytokines are not stored preformed in mast cells but are synthesised *de novo* and typically become detectable at later time points.

The immunogenicity data further support this interpretation. While PLA2-specific IgE levels remained largely unchanged following VIT, as often observed in human AIT studies (26), robust increases in allergen-specific IgG1 and IgG2b were induced in a dose- and formulation-dependent manner. These IgG subclasses are considered functional correlates of protection in murine models, as they can interfere with IgE-mediated allergen presentation and mast cell activation, thereby contributing to immune tolerance. Importantly, comparable IgG responses were also observed when using whole bee venom extract, indicating that the induced protective antibody response is not restricted to the major allergen PLA2 but extends to the broader allergen repertoire.

In murine models of allergy, both IgG1 and IgG2 subclasses are widely regarded as indicators of protective immunity, comparable to the potential predictive value of IgG4 in humans (27); notably, mice do not produce IgG4. While not directly homologous to human IgG4, murine IgG1 and IgG2 share functional properties with human blocking antibody responses observed during AIT, including the ability to interfere with IgE-mediated effector mechanisms (28, 29). Although no clear consensus exists as to whether IgG1 or IgG2 provides superior protection against allergic anaphylaxis, IgG1 is generally considered to act primarily through blocking functions, whereas IgG2 subclasses may additionally mediate protection via engagement of Fc*γ* receptors (30).

Safety is a critical consideration in the development of new VIT formulations. Given the risk of systemic reactions during VIT, we carefully monitored clinical signs and body temperature following treatment. Mild, transient reactions such as piloerection and limited hypothermia were observed predominantly after the first VIT injection and decreased markedly with subsequent administrations. Importantly, these reactions were most frequent in the aqueous VIT group and least frequent in the MCT-based group, with no evidence of severe or progressive systemic adverse events. These findings suggest that MCT does not increase, and may even reduce, the risk of VIT-associated systemic reactions in this model.

Mechanistically, the favourable profile of MCT-based VIT can be explained by two complementary properties. First, MCT acts as a depot-forming agent, limiting the immediate bioavailability of the allergen at the injection site and preventing rapid bolus release into the systemic circulation. Such bolus exposure may be a key driver of dose-dependent adverse reactions in VIT. By enabling slow and controlled allergen release, MCT improves safety while simultaneously providing prolonged antigen exposure that supports immunogenicity and treatment efficacy. Second, formulating allergens in a particulate form enhances uptake by professional antigen-presenting cells such as dendritic cells and macrophages. Improved phagocytic uptake facilitates antigen processing and presentation, thereby promoting efficient induction of protective adaptive immune responses and maintaining the immunogenicity of the native venom allergens.

These findings are consistent with previous studies on MCT-adjuvanted allergen immunotherapy in aeroallergen settings, where MCT has demonstrated favourable safety and efficacy profiles. Clinical studies and meta-analyses in grass pollen immunotherapy have shown that MCT-containing formulations induce robust protective immune responses while maintaining good tolerability, including in short-course treatment regimens (12, 31–34). Together, these data support the broader applicability of MCT as a depot adjuvant across different allergen systems.

In summary, our data demonstrate that MCT can be used as a depot adjuvant for venom immunotherapy without compromising efficacy or safety in a murine model of venom-induced anaphylaxis. MCT-adjuvanted VIT effectively induces protective IgG responses and limits mast cell activation upon allergen challenge similarly to aqueous and alum-adjuvanted VIT and reduces the frequency of systemic reactions during treatment. Together, these findings support further evaluation of MCT as a complementary adjuvant to aqueous venom extracts in venom immunotherapy.

## Data Availability

The raw data supporting the conclusions of this article will be made available by the authors, without undue reservation.

## References

[1] Sturm GJ Varga EM Roberts G Mosbech H Bilo MB Akdis CA EAACI guidelines on allergen immunotherapy: hymenoptera venom allergy. Allergy. (2018) 73(4):744–64. 10.1111/all.13262 28748641

[2] Dhami S Zaman H Varga EM Sturm GJ Muraro A Akdis CA Allergen immunotherapy for insect venom allergy: a systematic review and meta-analysis. Allergy. (2017) 72(3):342–65. 10.1111/all.13077 28120424

[3] Becker S Feindor M Graessel A Fernandez de Alba I Birkholz K Raab J Patient preference in allergen immunotherapy - understanding the patient’s view. World Allergy Organ J. (2025) 18(12):101154. 10.1016/j.waojou.2025.101154 41492423 PMC12765409

[4] Anton M Cabanes N Fernandez-Melendez S Fernandez-Nieto M Jimenez-Ferrera G Letran A Shared decision-making in allergen immunotherapy (AIT) options using a questionnaire for respiratory allergic patients: a delphi consensus study. Patient Prefer Adherence. (2023) 17:1771–82. 10.2147/PPA.S409466 37520065 PMC10378527

[5] Gehrt F Xu Q Baiardini I Canonica GW Pfaar O. Adherence in allergen immunotherapy: current situation and future implications. Allergol Select. (2022) 6:276–84. 10.5414/ALX02318E 36457724 PMC9707370

[6] Pravettoni V Mauro M Rivolta F Consonni D Cappelletti C Chiei Gallo A Venom immunotherapy: safety and tolerability of the build-up phase with depot versus aqueous preparations. Clin Exp Allergy. (2022) 52(10):1230–3. 10.1111/cea.14209 35904013 PMC9796821

[7] Incorvaia C Frati F Dell'Albani I Robino A Cattaneo E Mauro M Safety of hymenoptera venom immunotherapy: a systematic review. Expert Opin Pharmacother. (2011) 12(16):2527–32. 10.1517/14656566.2011.616494 21883032

[8] Kramer MF. Non-purified” hymenoptera venom extracts—from protein to preferred product. Allergo J Int. (2026) 35:22–7. 10.1007/s40629-025-00362-8

[9] Fischer J Loffelad L Kranert P Kneilling M Volc S. Impact of naturally composed and size-excluded hymenoptera venom preparations on the safety and efficacy of venom immunotherapy: a monocentric experience. Int Arch Allergy Immunol. (2025) 187(4):366–74. 10.1159/000547194 40706583 PMC12503586

[10] Cadario G Marengo F Ranghino E Rossi R Gatti B Cantone R Higher frequency of early local side effects with aqueous versus depot immunotherapy for hymenoptera venom allergy. J Investig Allergol Clin Immunol. (2004) 14(2):127–33. 15301302

[11] Leuthard DS Duda A Freiberger SN Weiss S Dommann I Fenini G Microcrystalline tyrosine and aluminum as adjuvants in allergen-specific immunotherapy protect from IgE-mediated reactivity in mouse models and act independently of inflammasome and TLR signaling. J Immunol. (2018) 200(9):3151–9. 10.4049/jimmunol.1800035 29592962 PMC5911931

[12] Jensen-Jarolim E Bachmann MF Bonini S Jacobsen L Jutel M Klimek L State-of-the-art in marketed adjuvants and formulations in allergen immunotherapy: a position paper of the European academy of allergy and clinical immunology (EAACI). Allergy. (2020) 75(4):746–60. 10.1111/all.14134 31774179

[13] Hiller J Goen T Drexler H Berking C Wagner N. Elevated aluminum excretion in patients by long-term subcutaneous immunotherapy - A cross-sectional case-control study. Int J Hyg Environ Health. (2024) 258:114337. 10.1016/j.ijheh.2024.114337 38461738

[14] Kramer MF Heath MD. Aluminium in allergen-specific subcutaneous immunotherapy–a German perspective. Vaccine. (2014) 32(33):4140–8. 10.1016/j.vaccine.2014.05.063 24892252

[15] Wahl RU Wurpts G Merk HF. Post-vaccination granulomas caused by delayed-type reaction to aluminum salts. Hautarzt. (2014) 65(5):384–6. 10.1007/s00105-014-2793-4 24736873

[16] Weisser K Wangorsch G Hartung N Huisinga W Keller-Stanislawski B. Model-informed exploration of the boundaries of safe aluminium exposure from allergen immunotherapy in children. Pediatr Allergy Immunol. (2025) 36(8):e70181. 10.1111/pai.70181 40856306 PMC12379567

[17] Lin YJ Zimmermann J Schulke S. Novel adjuvants in allergen-specific immunotherapy: where do we stand? Front Immunol. (2024) 15:1348305. 10.3389/fimmu.2024.1348305 38464539 PMC10920236

[18] Johnson L Duschl A Himly M. Nanotechnology-based vaccines for allergen-specific immunotherapy: potentials and challenges of conventional and novel adjuvants under research. Vaccines. (2020) 8(2):237. 10.3390/vaccines8020237 32443671 PMC7349961

[19] Heath MD Mohsen MO de Kam PJ Carreno Velazquez TL Hewings SJ Kramer MF Shaping modern vaccines: adjuvant systems using MicroCrystalline tyrosine (MCT). Front Immunol. (2020) 11:594911. 10.3389/fimmu.2020.594911 33324411 PMC7721672

[20] Cabral-Miranda G Heath MD Gomes AC Mohsen MO Montoya-Diaz E Salman AM Microcrystalline tyrosine (MCT): a depot adjuvant in licensed allergy immunotherapy offers new opportunities in malaria. Vaccines. (2017) 5(4):32. 10.3390/vaccines5040032 28953265 PMC5748599

[21] Percie du Sert N Hurst V Ahluwalia A Alam S Avey MT Baker M The ARRIVE guidelines 2.0: updated guidelines for reporting animal research. BMJ Open Sci. (2020) 4(1):e100115. 10.1136/bmjos-2020-100115 34095516 PMC7610906

[22] Paolucci M Homere V Waeckerle-Men Y Wuillemin N Bieli D Pengo N Strain matters in mouse models of peanut-allergic anaphylaxis: systemic IgE-dependent and ara h 2-dominant sensitization in C3H mice. Clin Exp Allergy. (2023) 53(5):550–60. 10.1111/cea.14279 36629248

[23] Paolucci M Wuillemin N Homere V Bieli D Kohli A Ballmer-Weber B Targeting ara h 2 with human-derived monoclonal antibodies prevents peanut-induced anaphylaxis in mice. Allergy. (2023) 78(6):1605–14. 10.1111/all.15659 36704937

[24] Khodoun MV Strait R Armstrong L Yanase N Finkelman FD. Identification of markers that distinguish IgE- from IgG-mediated anaphylaxis. Proc Natl Acad Sci U S A. (2011) 108(30):12413–8. 10.1073/pnas.1105695108 21746933 PMC3145724

[25] Storni F Vogel M Bachmann MF Engeroff P. Igg in the control of Fc*ε*RI activation: a battle on multiple fronts. Front Immunol. (2023) 14:1339171. 10.3389/fimmu.2023.1339171 38274816 PMC10808611

[26] Suhrkamp I Scheffold A Heine G. T-cell subsets in allergy and tolerance induction. Eur J Immunol. (2023) 53(10):e2249983. 10.1002/eji.202249983 37489248

[27] Jarkvist J Salehi C Akin C Gulen T. Venom immunotherapy in patients with clonal mast cell disorders: igG4 correlates with protection. Allergy. (2020) 75(1):169–77. 10.1111/all.13980 31306487

[28] Nagata Y Suzuki R. Insights into complex murine models of allergy and anaphylaxis: the central role of IgE and mast cells in advancing human therapies. Eur J Immunol. (2025) 55(12):e70117. 10.1002/eji.70117 41416944 PMC12716187

[29] Reber LL Hernandez JD Galli SJ. The pathophysiology of anaphylaxis. J Allergy Clin Immunol. (2017) 140(2):335–48. 10.1016/j.jaci.2017.06.003 28780941 PMC5657389

[30] Bruhns P Jonsson F. Mouse and human FcR effector functions. Immunol Rev. (2015) 268(1):25–51. 10.1111/imr.12350 26497511

[31] Zielen S Bernstein JA Sturm GJ Jutel M Pfaar O Shamji MH Six injections of modified adjuvanted PQ grass is effective and well-tolerated in a pivotal phase III trial. Allergy. (2025) 80(7):1982–94. 10.1111/all.16491 39905623 PMC12261879

[32] Al Saleh H Mosges R. Microcrystalline tyrosine-adsorbed immunotherapy. Curr Opin Allergy Clin Immunol. (2022) 22(6):413–20. 10.1097/ACI.0000000000000859 36254926 PMC9944405

[33] Becker S Zieglmayer P Canto G Fassio F Yong P Acikel C A meta-analysis on allergen-specific immunotherapy using MCT((R)) (MicroCrystalline tyrosine)-adsorbed allergoids in pollen allergic patients suffering from allergic rhinoconjunctivitis. Clin Transl Allergy. (2021) 11(4):e12037. 10.1002/clt2.12037 34523256 PMC8174800

[34] Zielen S Bernstein JA Pfaar O DuBuske L Mosges R Becker S Meta-analysis of PQ grass 27600 SU efficacy and quality of life from phase III trials. Allergy. (2025) 80(7):2055–8. 10.1111/all.16535 40162598 PMC12261871

